# Assessment of recovery in older patients hospitalized with different diagnoses and functional levels, evaluated with and without geriatric assessment

**DOI:** 10.1186/s11556-016-0166-y

**Published:** 2016-06-01

**Authors:** Jenny Foss Abrahamsen, Cathrine Haugland, Anette Hylen Ranhoff

**Affiliations:** Department of nursing home medicine, Municipality of Bergen and Kavli Research Centre for Geriatrics and Dementia, Haraldsplass Deaconess Hospital, Ulriksdal 8, 5009 Bergen, Norway; Storetveit nursing home, Kirkeveien 29, 5072 Bergen, Norway; Kavli Research Centre for Geriatrics and Dementia, Haraldsplass Deaconess Hospital, Ulriksdal 8, 5009 Bergen, Norway; Departement of Clinical Science, University of Bergen, Bergen, Norway

**Keywords:** Geriatric assessment, Recovery, Post-acute care, Barthel index, Hospitalization

## Abstract

**Background:**

The objective of the present study was to investigate 1) the role of different admission diagnoses and 2) the degree of functional loss, on the rate of recovery of older patients after acute hospitalization. Furthermore, to compare the predictive value of simple assessments that can be carried out in a hospital lacking geriatric service, with assessments including geriatric screening tests.

**Methods:**

Prospective, observational cohort study, including 961community dwelling patients aged ≥ 70 years, transferred from medical, cardiac, pulmonary and orthopedic acute hospital departments to intermediate care in nursing home. Functional assessment with Barthel index (BI) was performed at admission to the nursing home and further geriatric assessment tests was performed during the first week. Logistic regression models with and without geriatric assessment were compared concerning the patients having 1) slow recovery (nursing home stay up to 2 months before return home) or, 2) poor recovery (dead or still in nursing home at 2 months).

**Results:**

Slow recovery was independently associated with a diagnosis of non-vertebral fracture, BI subgroups 50–79 and <50, and, in the model including geriatric assessment, also with cognitive impairment. Poor recovery was more complex, and independently associated both with BI < 50, receiving home care before admission, higher age, admission with a non-vertebral fracture, and in the geriatric assessment model, cognitive impairment.

**Conclusions:**

Geriatric assessment is optimal for determining the recovery potential of older patients after acute hospitalization. As some hospitals lack geriatric services and ability to perform geriatric screening tests, a simpler assessment based on admission diagnoses and ADL function (BI), gives good information regarding the possible rehabilitation time and possibility to return home.

## Background

Functional decline and increasing dependency may occur when older patients hospitalized for acute disease or injury. Several studies have addressed this, but most of these studies include patients with either a medical [1–3], orthopaedic [4], or subgroups of diagnoses such as hip fracture [5] or stroke [6]. To our knowledge, no studies have analysed a mixed population from different hospital departments, to assess the influence of different admission diagnoses on functional recovery after acute hospitalization.

Comprehensive geriatric assessment, CGA, is considered the optimal way to assess older people admitted acutely to hospital [7]. CGA predicts mortality and adverse outcomes in hospitalized geriatric patients [8], and there is strong evidence in the literature that comprehensive geriatric care (CGC) improves outcomes for older people after acute hospitalization [9, 10]. However, in clinical daily life, a majority of older patients are admitted to hospitals or institutions lacking geriatric service and the ability to perform a full CGA. Thus focus on simple assessments tools, like the Barthel Index sumscore (hereafter called BI), is still important to elaborate on.

In 2005, a 19 bed post-acute intermediate care (IC) unit, based on CGC and increased multidisciplinary staffing, was introduced in our nursing home. The aim was to provide treatment and rehabilitation for elderly people within a few days after acute hospitalization. Emphasis was put on selecting patients that would need a short treatment and/or rehabilitation stay, to allow a rather high turnover of patients. In two previous studies from this IC unit, orthopaedic diagnosis and increasing functional dependence, assessed with BI, were associated with an unfavourable short term clinical outcome after acute hospitalization [11, 12]. Similar association between BI and rate of recovery has been demonstrated in several other reports [11, 13–16].

The primary objective of the present study was to investigate the role and predictive value of 1) different admission diagnoses and 2) the degree of functional loss, assessed by BI, on the recovery potential of older patients after acute hospitalization. Furthermore, to assess the predictive value of 1) a simple assessment that could be carried out in a hospital or facility lacking geriatric service, and 2) a more comprehensive assessment including specialized geriatric tests. Emphasis was put on characterizing the patients that experienced an unsuccessful recovery, to gain more knowledge regarding future post-acute care models.

## Methods

### Design and setting

This study is part of a prospective, observational, cohort study that during the 3 years 2011–2014, enrolled 961 consecutive patients 70 years or older after acute admissions to the two hospitals in Bergen. The design, setting and patients have been described more extensively in two other studies [11, 12].

After a short stay of median 5 days in the hospital for establishing the diagnoses and start of therapy, the patients were transferred to the nursing home 19-bed IC unit. The staffing was approximately to the level of a community hospital with increased multidisciplinary personnel (two fulltime physicians, one of them being a geriatrician, 15 nurses, 1.2 positions for physiotherapists, and 0.8 positions for an occupational therapist). If the patient could not return home within 14 days, transfer to an ordinary lower-cost, skilled nursing facility should occur. In these premises, the multidisciplinary staffing was approximately 1/3 of the staffing in the IC unit.

### Patients

The inclusion criteria were as follows:

1. The patients were ≥ 70 years of age, home-dwelling in the municipality of Bergen and considered to be respiratory and circulatory stable.
2. The hospital doctor expected that the patients would be able to return home within 2 weeks of treatment in the IC unit.
3. The patients did not have a major cognitive impairment or delirium, (based on the clinical judgement by the hospital doctor).

The patients that were transferred to the IC unit comprised approximately 20 % of all admissions from hospital to nursing homes in the municipality of Bergen. Both medical patients (from the departments of internal medicine, including cardiology and pulmonology) and orthopaedic patients were admitted. Most of the orthopaedic patients had suffered a fall, and none were admitted after elective surgery.

### Subdivision of patients into rapid, slow and poor recovery groups

Patients were divided into 3 groups based on their ability to return home after acute hospitalization and nursing home stay [12].

The rapid recovery group included the patients who were able to return directly home from the short term stay (median 14 days, range 2–31) in the IC unit. Follow-up of these patients demonstrated that 87 % of them were living at home 6 months after the acute hospitalization [12].

The slow recovery group included patients who were not able to return home after a short term stay in the IC unit, and had to be transferred to an ordinary skilled nursing facility. However, 2 months after the acute hospitalization, these patients had returned to their own home, and 6 months after hospitalization, 87 % of them were still living at home [12].

The poor recovery group included patients who were not able to return home after short-term nursing home IC and were transferred to an ordinary skilled nursing facility. Two months after hospitalization these patients were either dead or still living in a nursing home. Six months after hospitalization, only 20 % of them were living at home [12].

### Data collection and subgrouping of Barthel Index and diagnoses into 3 different groups

The data on patient’s demographic and baseline clinical characteristics were obtained from hospital records.

In 99 % of the patients, functional capacity was assessed by BI at admission by nurses in the IC unit observing the patients [17]. BI scores 10 different ADL-items (feeding, bathing, grooming, dressing, defecation, bladder function, ability to use the toilet, transfer, mobility and climbing stairs). The score range is 0–100, the highest score indicates the best function. Patients with different BI scores were divided into three meaningful clinical groups: Severely decreased ADL: BI < 50, moderate reduced ADL: BI 50–79, and independent ADL: BI 80–100, according to another study reporting on 5087 geriatric patients [15].

**Fig. 1:**
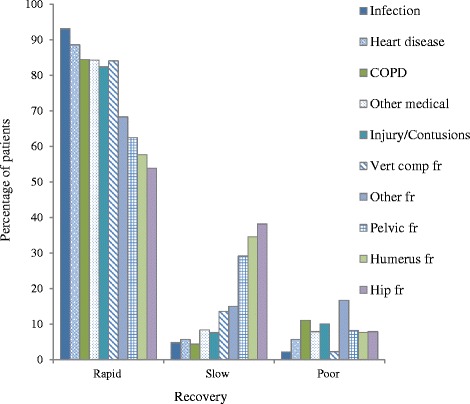
Rapid, slow and poor recovery in patients with different diagnoses

Patients with different diagnoses were divided into three meaningful clinical groups depending on the rate of recovery, as indicated in Fig. 1. One subgroup included the patients from the medical, pulmonology and cardiac departments. A second subgroup included patients from both the medical and orthopaedic department with minor trauma/contusions and vertebrae compression fractures. The third subgroup included patients from the orthopaedic department with all other fractures, including 76 hip fractures.

In addition to BI, further geriatric assessment was performed during the first week on >90 % of the patients by the following geriatric screening tests: 1) The Norwegian version of the Mini Mental Status Examination, MMSE [18, 19]. (Score range 0–30, higher scores indicate better cognitive status and score < 24 is considered a sign of cognitive impairment. 2) Geriatric Depression Scale, GDS [20] score range 0–30, higher score indicates increasing symptoms of depression) and 3) Mini Nutritional Assessment - Short Form; MNA-SF [21]. Lower score indicates malnutrition and a score < 8 gives suspicion of malnutrition. Patients with suspected delirium on admission to the IC unit were evaluated by the nursing home geriatrician using the Confusion assessment method (CAM).

Information on whether the patients returned home after transfer to an ordinary nursing home, residence status and survival, was obtained from the patient administrative system in the municipality.

### Statistical analyses

For identifying the clinical characteristics that were associated with a rapid, slow and poor recovery, odds ratios (ORs) with 95 % confidence intervals (CIs) were estimated using logistic regression models. The patients with a rapid recovery were compared to the rest of the patients having a slow or poor recovery. Each of the patient groups with slow or poor recovery was compared with the patients that had a rapid recovery.

The characteristics associated with *p* < 0.25 in univariate analysis were noted as likely predictors and included in multivariate, adjusted logistic regression models. The characteristics associated with *p* ≤ 0.05 was considered statistically significant in the multivariate models. Two logistic multivariate regression models were analyzed; 1) a model including BI and further geriatric tests; MMSE, GDS and MNA (geriatric model) and 2) a simpler model not including MMSE, GDS and MNA (Table 2).

The analyses were performed using the Statistical Package for Social Science (IBM SPSS), version 20 for Windows.

## Results

### Patient characteristics

Of 1085 patients who were admitted to the IC unit during the period 2011–2014, 112 were not asked to participate in the study at times when the geriatrician in charge was absent, 5 patients refused to participate, 4 patients had delirium and 3 patients had language problems. Thus, altogether 961 patients were included in the study.

Table 1 shows the baseline clinical patient characteristics of the whole patient population as well as subgroups of patients according to different diagnoses groups. Differences were demonstrated between patients in the three major diagnostic groups, primarily concerning BI scores and clinical outcome.

**Table 1:** Characteristics of all patients transferred to post-acute care and subdivision into three different diagnostic groups

		All diagnoses *N* = 961	Medical *N* = 609	Contusion/vert comp fr, *n* = 165	Fractures *N* = 187
	n^a^		Infection (231) Heart (141) COPD (45) Other (192)	Contusion (121) Vert comp fr (44)	Hip (76) Humerus (26) Pelvis (25) Other (60)
Clinical variables					
BI at admission, med (min-max)	954	75 (10–100)	80 (25–100)	75 (25–100)	60 (10–100)
BI 80–100, n (%)		449 (47)	344 (59)	64 (39)	29 (16)
BI 50–79, n (%)		418 (44)	215 (36)	85 (52)	119 (64)
BI <50, n (%)		170 (29)	33 (5)	15 (9)	39 (21)
Age, med (min-max)	961	85 (70–102)	85 (70–102)	86 (70–100)	83 (70–98)
Female sex, n (%)	961	656 (68)	397 (65)	123 (74)	135 (72)
>5 diagnoses, n (%)	910	567 (62)	394 (68)	89 (57)	84 (48)
Use >5 drugs, n (%)	947	760 (80)	499 (83)	128 (80)	133 (72)
Live alone, n (%)	912	644 (71)	399 (70)	126 (81)	119 (65)
Receive home care, (%)	953	379 (39)	256 (42)	73 (46)	47 (26)
Admitted after a fall, (%)	947	382 (40)	93 (15)	119 (73)	172 (93)
Geriatric screening					
MMSE, med (min-max)	858	26 (8–30)	26 (8–30)	26 (11–30)	26 (12–30)
MMSE < 24, n (%)	858	206 (29)	153 (29)	45 (31)	51 (29)
GDS, med (min-max)	851	7 (0–29)	7 (0–29)	7 (0–26)	7 (0–28)
MNA-SF, n (%)	912	10 (2–21)	10 (2–19)	10 (4–16)	10 (3–21)
Outcome					
Rapid recovery, n (%)	957	785 (82)	535 (89)	135 (82)	112 (60)
Slow recovery, n (%)	957	106 (11)	38 (6)	15 (9)	54 (29)
Poor recovery, n (%)	957	66 (7)	33 (5)	11 (9)	20 (11)

*med* median, *Vert comp fr*, vertebrae compression fractures, *Heart* heart disease including ischemic heart disease and cardiac failure, *COPD* chronic obstructive pulmonary disease, *ADL* Activities of Daily Living, *BI* Barthel index, *MMSE* Mini-Mental Status Examination, *GDS* Geriatric Depression Scale (0–30), *MNA-SF* Mini Nutritional Assessment-Short Form

^a^number of patients examined

The different rate of recovery according to the ten different diagnoses groups are shown in Fig. 1. The figure indicates that the majority of patients with a medical diagnosis and contusion/injury diagnosis had rapid recovery. Slow recovery was more often observed for patients with non-vertebral fractures, while the influence of diagnoses was not so pronounced for the groups of patients that had a poor recovery.

### Variables associated with slow recovery

As shown in Table 2, only BI subgroups and diagnosis of a non-vertebral fracture, were independently associated with slow recovery, in the multivariate model with and without geriatric assessment. Cognitive impairment indicated in addition a significant, but rather weak odds ratio for slow recovery. When separate analyses were performed including all ten different diagnoses no particular fracture sub-group or any of the particular medical diagnoses were associated with a higher risk of slow recovery (*p* > 0.05).

**Table 2:** Multivariate regression models to predict recovery after acute hospitalization and post-acute care

	Univariate, unadjusted model	Multivariate, adjusted models	
		BI, no further geriatric assessment	BI and further geriatric assessment
	OR^a^ (95 % CI) *p*	OR (95 % CI) *p*	OR (95 % CI) *p*
RAPID recovery^a^, *n* = 785			
BI 80–100 (ref)			
BI 50–79	0.24 (0.16–.0.37) <0.001	0.31 (0.23–0.58) <0.001	0.35 (0.19–0.62) <0.001
BI < 50	0.07 (0.04–0.13) <0.001	0.16 (0.08–0.29) <0.001	0.17 (0.08–0.37) <0.001
Medical diagnosis (ref)			
Contusions/vert comp fr	0.62 (0.38–1.00) 0.05	0.69 (0.38–1.25) 0.22	0.63 (0.29–1.24) 0.17
All other fractures	0.19 (0.13–0.29) <0.001	0.21 (0.11–0.39) <0.001	0.15 (0.07–0.31) <0.001
Age^d^	0.97 (0.94–0.99) 0.01	0.97 (0.94–0.99) 0.02	0.98 (0.95–1.02) 0.27
Receive home care	0.61 (0.44–0.86) 0.004	0.64 (0.42–0.95) 0.03	0.88 (0.53–1.45) 0.61
Admitted after a fall	0.38 (0.27–0.54) <0.001	0.84 (0.49–1.44) 0.53	0.75 (0.39–1.44) 0.39
MMSE < 24	0.35 (0.24–0.50) <0.001	__	0.46 (0.29–0.74) <0.001
GDS^d^	0.95 (0.92–0.98) <0.001	__	0.95 (0.91–0.98) 0.004
MNA-SF^d^	1.06 (1.00–1.13) 0.06	__	0.99 (0.91-1.07) 0.72
SLOW recovery^b^, *n* = 106			
BI 80–100 (ref)			
BI 50–79	5.25 (3.03–9.12) <0.001	3.66 (1.90–7.05) <0.001	3.60 (1.86–6.96) <0.001
BI < 50	11.76 (5.78–23.95) < 0.001	6.30 (2.97–13.56) <0.001	4.16 (1.60–10.77) 0.003
Medical diagnosis (ref)			
Contusions/vert comp fr	1.61 (0.86–3.02) 0.14	1.35 (0.65–2.82) 0.42	0.90 (0.40–2.06) 0.81
All other fractures	6.92 (4.35–11.02) <0.001	4.50 (2.24–9.05) <0.001	6.58 (2.87–14.99) <0.001
Age^d^	1.00 (0.97–1.03) 0.99	__	__
Receive home care	0.96 (0.63–1.46) 0.85	__	__
Admitted after a fall	3.35 (2.18–5.15) <0.001	1.01 (0.53–1.94) 0.98	0.87 (0.41–1.84) 0.72
MMSE < 24	2.03 (1.28–3.24) 0.003	__	1.96 (1.12–3.42) 0.02
GDS^d^	1.04 (1.00–1.07) 0.04	__	1.04 (0.99–1.08) 0.07
MNA-SF^d^	0.98 (0.91–1.06) 0.65	__	__
POOR recovery^c^, *n* = 66			
BI 80–100 (ref)			
BI 50–79	2.72 (1.38–5.36) 0.004	1.87 (0.91–3.82) 0.09	2.52 (0.95–6.72) 0.06
BI < 50	16.02 (7.57–34.13) <0.001	6.87–2.94–16.01) <0.001	8.33 (2.76–25.16) <0.001
Medical diagnosis (ref)			
Contusions/vert comp fr	1.68 (0.88–3.23) 0.12	1.81 (0.75–4.31) 0.19	2.27 (0.96–5.38) 0.06
All other fractures	2.73 (1.50–4.97) 0.001	3.15 (1.20–8.25) 0.02	3.26 (1.04–10.21) 0.04
Age^d^	1.10 (1.05–1.15) <0.001	1.08 (1.02–1.31) 0.004	1.05 (0.99–1.17) 0.09
Receive home care	3.90 (2.23–6.79) <0.001	3.17 (1.64–6.15) 0.001	2.45 (1.07–5.62) 0.03
Admitted after a fall	1.73 (1.04–2.87) 0.04	0.73 (0.32–1.67) 0.46	0.92 (0.35–2.41) 0.87
MMSE < 24	5.02 (2.80–9.00) <0.001	__	2.95 (1.43–6.08) 0.003
GDS^d^	1.08 (1.04–1.13) <0.001	__	1.06 (1.00–1.13) 0.06
MNA-SF^d^	0.88 (0.80–0.97) 0.01	__	0.95 (0.83–1.01) 0.47

*BI* Barthel Index sumscore, *OR* Odds Ratio, *CI* confidence interval, *ref* reference category, *vert comp fr* vertebrae compression fracture, *MMSE* Mini Mental Status Examination, *GDS* Geriatric Depression Scale, *MNA-SF* Mini Nutritional Assessment-Short Form

^a^Return home within 2 weeks, ^b^Nursing home stay up to 2 months before return home, ^b^Dead or still in nursing home at 2 months, ^d^Variables are per unit increase

The variables sex, living alone, > 5 diagnoses and using > 5 drugs, demonstrated no association with slow or poor recovery in the univariate analysis, were not included in the multivariate analysis and are not shown in the table

### Variables associated with poor recovery

As shown in Table 2, in the multivariate analysis, only a severely reduced ADL (BI < 50), together with receiving homecare, diagnosis of a non-vertebral fracture, increasing age, and in the model with geriatric assessment, cognitive impairment, were significantly associated with poor recovery. No particular fracture subgroup or medical diagnosis was associated with a higher risk of poor recovery when this was analyzed in a separate multivariate analysis (*p* > 0.05). This is also indicated in Fig. 1.

### The effect of Barthel Index, diagnoses and cognitive impairment on the likelihood of slow and poor recovery, in multivariate regression analysis

As shown in Table 2, patients with a severe BI reduction (<50), were 6.9 and 8.3 times more likely to have poor recovery (models without and with geriatric assessment), and 6.3 and 4.2 more likely to have a slow recovery. Patients with a moderate BI reduction (50–79) were 3.7 and 3.6 more likely to have a slow recovery, but no more likely to have a poor recovery.

Patients with diagnosis of non-vertebral fractures were 4.5 and 6.6 times more likely to have a slow recovery but less likely (3.2 times) to have a poor recovery.

Cognitive impairment (MMSE <24) was most strongly associated with poor recovery (3 times increased odds), but also with slow recovery (2 times increased odds).

## Discussion

The present study demonstrates that slow recovery after acute hospitalization was independently associated with both moderate and severe ADL reduction (BI subgroups 50–79 and < 50), diagnosis non-vertebral fracture, and in the geriatric model, also with cognitive impairment. Poor recovery was more complex and independently associated with BI < 50, diagnosis of non-vertebral fracture, higher age, receiving home care, and in the CGA model, cognitive impairment.

It may be assumed that BI measured at arrival to the IC unit is corresponding to the BI that would have been measured at hospital discharge. The strong association between BI score and rate of recovery demonstrated here is in accordance with several other reports [11, 13–16]. The complex assessment of the risk of having a poor recovery after acute hospitalization is in accordance with a literature review of Campbell et al. which confirms this, and concludes that both functional and cognitive status affect the outcome in older hospitalized medical patients [22]. In line with our results, a recent review article also concludes that various factors (functional and cognitive status, age, ethnicity and depression) are significantly associated with home discharge after inpatient rehabilitation of older non-stroke patients [23].

A clinical implications of including BI assessments together with the conventional clinical assessment, is that more general information about further care after the acute hospitalization care can be achieved for the patients who cannot return directly home. Three different possible nursing home care pathways has recently been suggested [12], which can be further specified according to the present study: 1) Patients that based on clinical judgement, have a high probability of a rapid recovery, a BI score > 80, no fracture diagnosis, but still in need of further medical treatment or rehabilitation, could be offered short-term post-acute care in a skilled nursing facility with increased medical resources, as outlined in the present study. 2) Patients with a diagnosis of fracture or patients with a decreased BI < 80 could be considered for care in a skilled nursing facility with the likelihood for a longer treatment period and main focus on rehabilitation. 3) Patients with BI score < 50, high age, already receiving nurse assisted home care, and with suspected cognitive impairment, may be considered for care in a skilled nursing facility, with main emphasis on care and palliation. However, the latter group of patients is complicated to assess, and choosing the optimal management for them may require a multidisciplinary geriatric approach. These patients would possibly benefit from CGA during or sometime after the acute hospitalization, to assess the future recovery potential.

The major weakness of the present study is that only patients who were considered to need short term medical treatment and/or rehabilitation were included, and therefore the numbers of frail patients with severe physical impairment, belonging mainly to the poor recovery groups, is low. The patients were recruited from the same area and treated in a single institution, thus the generalizability of the study may be limited. Furthermore, the results of the present study cannot be used to tailor IC to individual patients. The strength of the study is that BI was performed on nearly all of the patients, and we were able to compare simple screening with more advanced geriatric screening concerning two unfavourable recovery outcomes. Contrary to most other studies that include smaller subgroups of patients, the present study includes all of them and thus the influence of different diagnosis groups on the recovery potential could be investigated. In practical terms, these patients share many clinical characteristics, are admitted to the same emergency department, and are cared for in the same type of facilities.

## Conclusion

Geriatric assessment is optimal for determining the recovery potential of older patients after acute hospitalization. However, some hospitals or institutions lack geriatric services and ability to perform a full CGA. The present study shows that in these cases, a simpler and easy accessible assessment, based on ADL function (BI) and diagnoses, give good information regarding the possible rehabilitation time and possibility to return to own home after acute hospitalization.
